# Phosphatidylinositol 3-kinase (PI3Kα)/AKT axis blockade with taselisib or ipatasertib enhances the efficacy of anti-microtubule drugs in human breast cancer cells

**DOI:** 10.18632/oncotarget.20385

**Published:** 2017-08-22

**Authors:** Floriana Morgillo, Carminia Maria Della Corte, Anna Diana, Concetta di Mauro, Vincenza Ciaramella, Giusi Barra, Valentina Belli, Elisena Franzese, Roberto Bianco, Evaristo Maiello, Ferdinando de Vita, Fortunato Ciardiello, Michele Orditura

**Affiliations:** ^1^ Oncologia Medica, Dipartimento di Internistica Clinica e Sperimentale “F. Magrassi”, Università degli Studi della Campania “Luigi Vanvitelli”, Napoli, Italy; ^2^ Oncologia Medica, Dipartimento di Medicina Clinica e Chirurgia, Università degli Studi di Napoli “Federico II”, Napoli, Italy; ^3^ Immunologia Clinica, Dipartimento di Internistica Clinica e Sperimentale “F. Magrassi”, Università degli Studi della Campania “Luigi Vanvitelli”, Napoli, Italy; ^4^ IRCCS Casa Sollievo della Sofferenza, San Giovanni Rotondo, Foggia, Italy

**Keywords:** AKT, breast cancer, taselisib, ipatasertib, novel drugs

## Abstract

**Purpose:**

The Phosphatidylinositol 3-kinase (PI3Ks) pathway is commonly altereted in breast cancer patients, but its role is still unclear. Taselisib, a mutant PI3Kα selective inhibitor, and ipatasertib, an AKT inhibitor, are currently under investigation in clinical trials in combination with paclitaxel or hormonal therapies in breast cancer. The aim of this study was to evaluate if PI3K or AKT inhibition can prevent resistance to chemotherapy and potentiate its efficacy.

**Experimental design:**

The efficacy of combined treatment of ipatasertib and taselisib plus vinorelbine or paclitaxel or eribulin was evaluated *in vitro* on human breast cancer cells (with different expression profile of hormonal receptors, HER2, and of PI3Ka mutation) on cell survival by using MTT (3,(4,5-dimethylthiazol-2)2,5 difeniltetrazolium bromide) and colony forming assays on cell apoptosis by flow-cytometry analysis. We also investigated the effect of combined treatment on downstream intracellular signaling, by western blot analysis, and on metastatic properties, by migration assays. Finally, we analyzed changes in cell cytoskeleton by immunofluorescence.

**Results:**

A significant synergism of ipatasertib or taselisib plus anti-microtubule chemotherapy in terms of anti-proliferative, pro-apoptotic and anti-metastatic effect was observed. The combined treatment completely inhibited the activation of proteins downstream of PI3K and MAPK pathways and affected the expression of survivin. Combined treatments completely disorganized the cytoskeleton in human breast cancer cells, with contemporary delocalization of survivin from cytoplasm to nucleus, thus suggesting a potential mechanism for this combination.

**Conclusions:**

Targeting PI3K may enhance the efficacy of anti-microtubule drugs in human breast cancer cells.

## INTRODUCTION

Breast cancer (BC) is the most frequent cancer among women with an estimated 1.6 million new cancer cases diagnosed in 2012 [1]. BC is a heterogeneous disease and is generally classified according to stage, hormone receptor (HR: estrogen receptor [ER] and/or progesterone receptor [PR]), and Human Epidermal Growth Factor Receptor 2 (HER2), which guide treatment decisions [2, 3].

Despite the recently observed reduction in mortality due to early diagnosis and improvement of treatment strategies, BC continues to be the leading cause of neoplastic death in women, regardless of age, and long-term survival of metastatic breast cancer (MBC) patients remains 43–50 months for HER2 positive disease and 30–45 months for HER2 negative and HR positive subtypes [4, 5].

Modern cancer therapies for breast cancer include chemoterapeutic, anti-hormonal and molecular agents [6–9]. Different classes of chemoterapic agents are currently approved for treatment of metastatic BC: antracylines, anti-mitotic agents (including taxanes, vinorelbine, eribulin), alkylating agents (such as cisplatin and carboplatin) and anti-metabolites (5-fluorouracile, capecitabine, and gemcitabine) [7–9].

More recently, research has been focused on mechanisms of resistance to conventional therapies in terms of intrinsic or acquired molecular defects involving intracellular signal transduction pathways in order to detect new targetable oncogenetic proteins [10].

The Phosphatidylinositol 3-kinase (PI3Ks) pathway is commonly altereted in breast cancer. PI3Ks pathway comprises a family of intracellular signal transducer enzymes with three key regulatory nodes: PI3K, AKT, and mammalian target of rapamycin (mTOR) [11]. Somatic mutations in the PI3K/AKT pathway genes have been identified with significant allelic frequencies in breast cancer, with PIK3CA being the most frequently altered in this tumor. In particular, somatic mutations are present in PIK3CA (36%), PIK3R1 (3%), PTEN (3%) and AKT1 (2%) genes (12). PIK3CA mutation frequencies are different among breast cancer subtypes: 34.5–45%, 22.7–39% and 8.3–25% in HR+, HER2+ and in triple negative breast cancers (TNBC), respectively [12–14]. About 90% of PIK3CA mutations, all missense, are located at hotspot clusters in the helical domain (HD) in exon 9 and kinase domain (KD) in exon 20. The activating mutations H1047R in the KD and E545K and E542K in the HD are the most prevalent alterations [15]. Noteworthy, PI3K/AKT signaling plays a key role in the pathogenesis of human breast cancer and has been hypothesized to confer resistance to systemic treatments including chemotherapy and HER2-targeted therapy [16, 17]. The relationship of PIK3CA mutations and AKT activation with prognosis and treatment benefit in human breast cancer represents an area of intense investigation with mixed results [18]. Furthermore, several studies have shown the association of PIK3CA mutations with the subsequent AKT activation status and have observed the modulation of AKT activity by chemotherapeutic agents and other cancer therapeutics [16, 17]. Thus, selective PI3K or AKT inhibitors represent a novel option to prevent resistance to chemotherapy and to potentially improve BC prognosis.

Taselisib [19–21], a novel selective inhibitor of mutant PI3Kα and ipatasertib (GDC-0068) [22–25], an inhibitor of all three AKT isoforms with a specific activity on mutant AKT1, are currently under development in phase II and III clinical trials in combination with paclitaxel or hormonal therapy in different setting of BC therapy (NCT02301988, NCT02162719, NCT02340221, NCT02273973, NCT01296555 and NCT01862081).

Here we have studied the efficacy of taselisib and ipatasertib in combination with different anti-microtubule chemotherapic agents by evaluating the anti-proliferative, pro-apoptotic and anti-migration effects and the cytoskeleton re-organization.

## RESULTS

### Synergistic effect of taselisib and ipatasertib in combination with chemotherapy in a panel of human breast cancer cell lines

To evaluate the antiproliferative effects of ipatasertib and taselisib as single agents or in combination with anti-microtubule chemotherapic agents, we used several human breast cancer cell models, including PI3Kα/Akt mutation negative and PI3Kα/Akt mutation positive cell lines, that have also different biologic profiles, according to the expression of HER2 and HR, as indicated in Table 1. MDA-MB231 and MDA-MB468 cells were selected as control, since they are human breast adenocarcinoma-derived cell lines harbouring *PI3K* wild-type gene. Among PI3Ka-mutated human breast cancer cell lines, we chose four cancer cell lines representative of each breast cancer subtype: BT474 cells (HER2/HR+), MCF7 (HR+), KPL4 (HER2+) and SUM159 (TNBC).

**Table 1:** Hystological and biological profile of the panel of breast cancer cell lines

Cell line	Hormonalreceptor status	HER2 expression	Disease
BT474	Positive	Positive	Ductal carcinoma
KPL-4	Negative	Positive	Adenocarcinoma
SUM159	Negative	Negative	Mesenchymal
MCF-7	Positive	Negative	Adenocarcinoma
MDA-MB231	Negative	Negative	Adenocarcinoma
MDA-MB468	Negative	Negative	Adenocarcinoma

1. Neve RM, Chin K, Fridlyand J, Yeh J, Baehner FL, Fevr T, Clark L, Bayani N, Coppe JP, Tong F, Speed T, Spellman PT, DeVries S, et al. A collection of breast cancer cell lines for the study of functionally distinct cancer subtypes. Cancer Cell. 2006; 10:515–27.

2. Subik K, Lee JF, Baxter L, Strzepek T, Costello D, Crowley P, Xing L, Hung MC, Bonfiglio T, Hicks DG, Tang P. The Expression Patterns of ER, PR, HER2, CK5/6, EGFR, Ki-67 and AR by Immunohistochemical Analysis in Breast Cancer Cell Lines. Breast Cancer (Auckl). 2010.

3. Kao J, Salari K, Bocanegra M, Choi YL, Girard L, Gandhi J, Kwei KA, Hernandez-Boussard T, Wang P, Gazdar AF, Minna JD, Pollack JR. Molecular profiling of breast cancer cell lines defines relevant tumor models and provides a resource for cancer gene discovery. PLoS One. 2009; 3;4:e6146.

Cell proliferation was measured with the 3-(4,5- dimethylthiazol-2-yl)-2,5 diphenyltetrazolium bromide (MTT) assay. As antimicrotubule agents, we selected paclitaxel, vinorelbine and eribulin, currently used for the treatment of metastatic breast cancer patients.

Different doses of ipatasertib, taselisib and anti-microtubules agents alone and in combination were tested. Cancer cell line characteristics and the IC_50_ values for the antiproliferative activity of each single drug are reported in Table 2. The IC_50_ values ranged from 10 nM to 500 nM for taselisib and from 0,5 mM to 10 mM for ipatasertib with the less sensitive cell line represented by the PI3K wild-type cell lines, MDA-MB468 and MDA-MB231. The IC_50_ values for the chemotherapic drugs ranged from 1 nM to > 100 nM.

**Table 2:** Mutational profile of the panel of breast cancer cell lines and IC_50_ doses for cell growth inhibition of single treatment with anti-microtubules chemotherapy, taselisib and ipatasertib

Cell line	PI3KCA status	IC50 Vinorelbine	IC50 Paclitaxel	IC50 Eribulin	IC50 Taselisib	IC50 Ipatasertib
BT474	p.K111N mutation	80 nM	10 nM	1 nM	10 nM	0,5 μM
KPL-4	p.H1047L mutation	150 nM	1 nM	2.5 nM	50 nM	0,5 μM
SUM159	p.H1047L mutation	150 nM	50 nM	5 nM	50 nM	2 μM
MCF-7	p.E545K mutation	100 nM	20 nM	2 nM	100 nM	1,5 μM
MDA-MB231	Wild-type	100 nM	2.5 nM	1 nM	500 nM	10 μM
MDA-MB468	Wild-type	100 nM	2.5 nM	1.5 nM	500 nM	10 μM

1. American Type Culture Collection (ATCC, Manassas, VA, USA).

2. COSMIC- Cell Lines Project- http://www.sanger.ac.uk/science/tools/cosmic.

**Figure 1:**
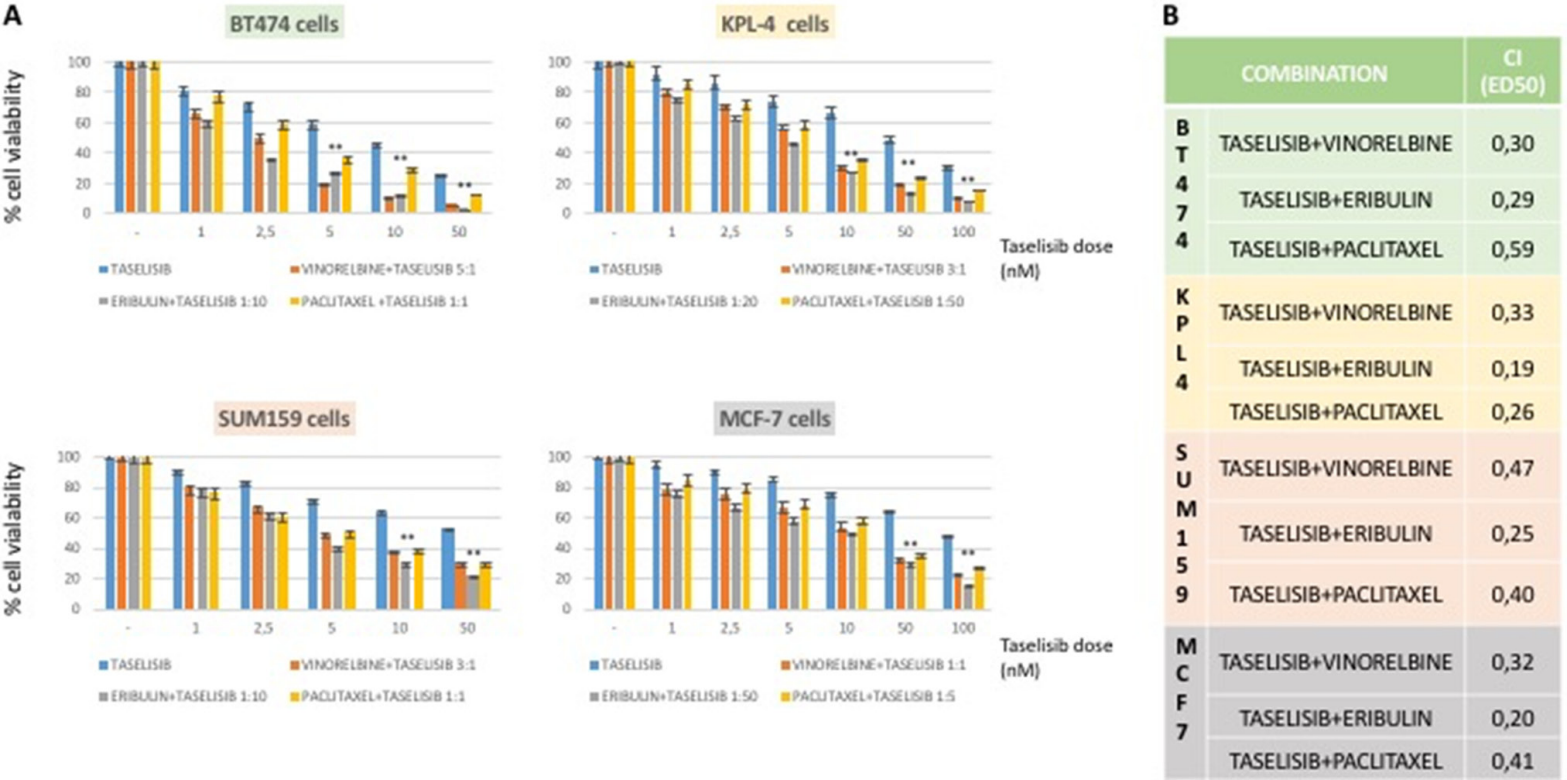
Effects on cell proliferation of taselisib treatment as single agent and combined with anti-microtubules chemotherapy in a panel of human BC cell lines (**A**) Cells were treated with different concentrations of taselisib and chemotherapy for 72 hours and evaluated for proliferation by MTT (3,(4,5-dimethylthiazol-2)2,5 difeniltetrazolium bromide) staining, as described in Materials and Methods. Constant ratio for combination was chosen considering the ratio between IC_50_ of each single drug. (**B**) Combination index (CI) was determined by CompuSyn analysis, for effect dose 50 (ED_50_) of each combination. Results represent the median of three separate experiments, each performed in quadruplicate. *P* values < 0.01 were considered as statistically significant (**).

**Figure 2:**
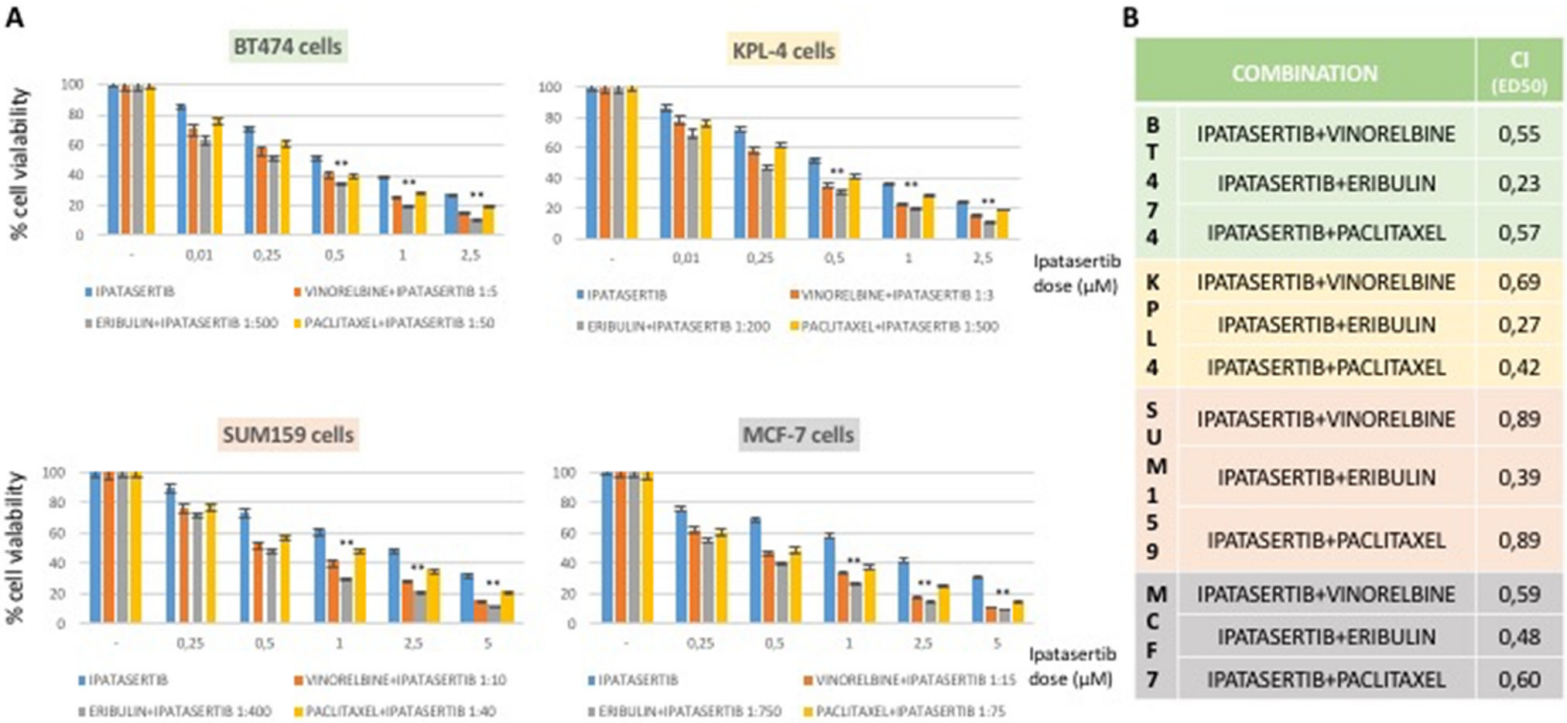
Effects on cell proliferation of ipatasertib treatment as single agent and combined with anti-microtubules chemotherapy in a panel of human BC cell lines (**A**) Cells were treated with different concentrations of ipatasertib and chemotherapy for 72 hours and evaluated for proliferation by MTT (3,(4,5-dimethylthiazol-2)2,5 difeniltetrazolium bromide) staining, as described in Materials and Methods. Constant ratio for combination was chosen considering the ratio between IC_50_ of each single drug. (**B**) Combination index (CI) CI was determined by CompuSyn analysis, for effect dose 50 (ED_50_) of each combination. Results represent the median of three separate experiments, each performed in quadruplicate. *P* values < 0.01 were considered as statistically significant (**).

Combined treatement of taselisib and antimicrotubule agents exerted a strong antiproliferative effect as compared to single treatment alone (Figure 1A) in PI3Kα-mutated breast cancer cells, with minor effect on the antoproliferative activity of eribulin, vinorelbin or paclitaxel in PI3Kα-wild-type MDA-MB468 and MDA-MB231 cell lines (data not shown). Similar results were obtained by the combined treatement of ipatasertib and anti-microtubules agents as compared to single treatment alone (Figure 2A) with the PI3Kα-mutated breast cancer cells resulting the most sensitive.

To quantify the effect of the combined therapy, we used the CompuSyn software to calculate the CI in all breast cancer cell lines. Sensitive cell lines had a CI index < 1 indicating synergism, according to the method of Chou-Talalay, using costant-ratio in each combination treatment (Figures 1B, 2B). No cell line showed an antagonistic effect by the combination therapies.

To confirm the anti-proliferative ability of these combinations, we performed colony forming assays and we obtained similary results (Supplementary Figure 1).

### Effect of taselisib and ipatasertib in combination with anti-microtubule chemotherapies on the induction of apoptosis in human breast cancer cell lines

**Figure 3:**
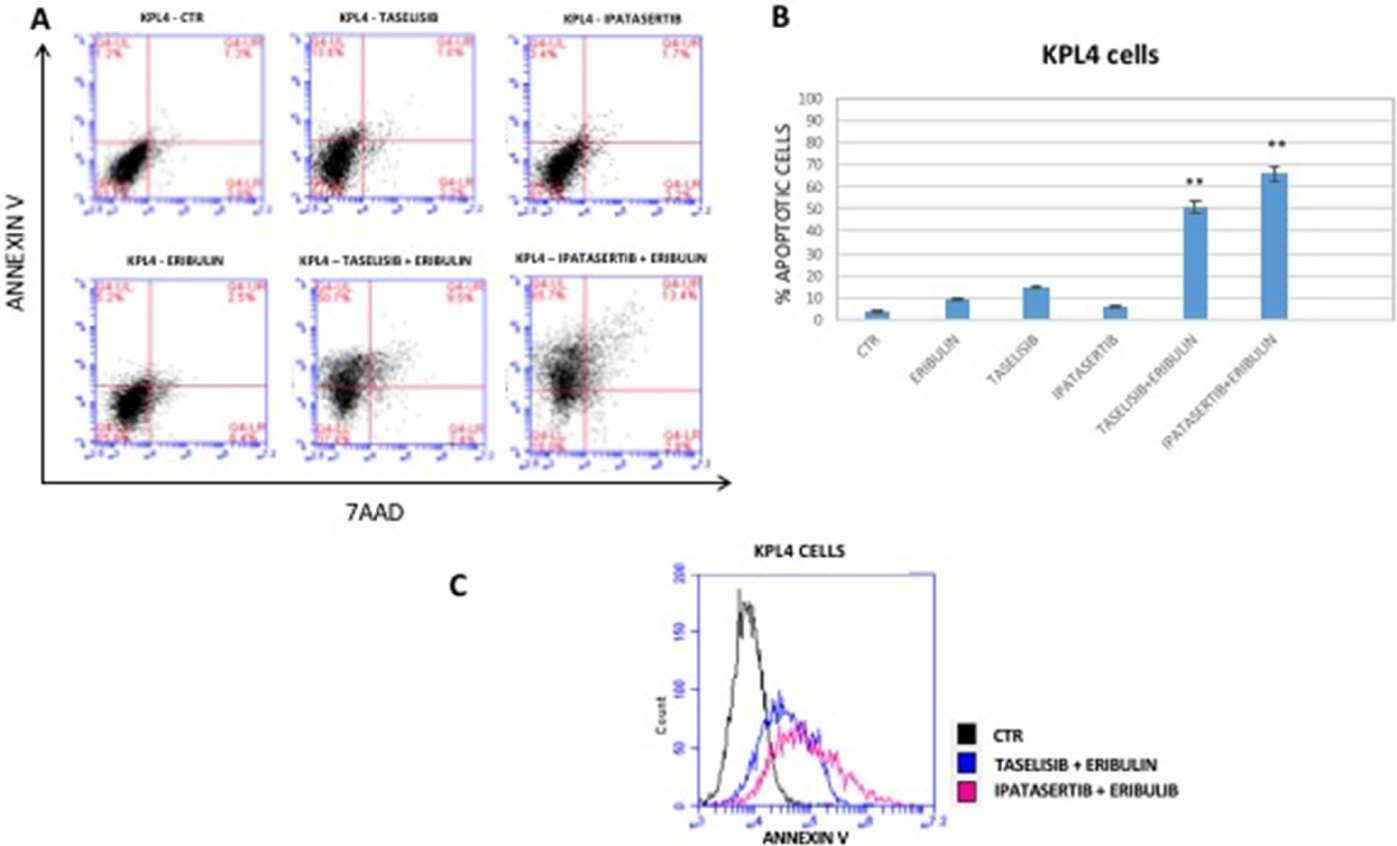
(**A**) Representative flow cytometric analysis of KPL4 cell apoptosis. One representative experiment is shown. Dot plots diagrams show the different stages of apoptosis. % indicated in the UL (Upper Left) quadrant represent cells positive for Annexin V and negative for 7AAD, considered as apoptotic cells; % in UR (Upper Right) quadrant indicate cells positive for both Annexin V and 7AAD, showing the late apoptotic or necrotic cells population; % in LL (Lower Left) quadrant are negative for both markers and represent viable cells. (**B**) Graphic representation of apoptosis in KPL4 cells. Bars represents mean values obtained from three separate experiments. (**C**) Histogram plot representing Annexin V positive KPL4 cells treated with the combination of drugs. *P* values < 0.01 were considered as statistically significant (**).

We next analyzed the induction of apoptosis in BT474, SUM159, MCF7 and KPL4 human breast cancer cell lines after 72-hour of treatment with taselisib or ipatasertib combined with either vinorelbine or eribulin. As shown in Figure 3A, flow cytometric analysis revealed that combined treatment with taselisib or ipatasertib with each anti-microtubule agent significantly increased of several folds the percentage of apoptotic cells in all cell lines tested. For instance, KPL4 cells presented respectively a 10,6%, 3,4% and 5,2% apoptotic rate in taselisib-, ipatasertib- and eribulin-treated cells (at single doses of 5nM, 250 nM and 0,5 nM, respectively), while the combination treatments reached an apoptotic rate of 50,7% and 65,7% apoptotic cells with eribulin plus taselisib or ipatasertib, respectively (Figure 3B). Figure 3C shows histogram plot representing Annexin V positive KPL4 cells treated with the combination of drugs.

Similar effects have been obtained in the other BC cell models (Supplementary Figure 2). Similar results have been obtained with the addition of taselisib or ipatasertib to paclitaxel or vinorelbine (data not shown).

### Effects of taselisib and ipatasertib in combination with anti-microtubule drugs on intracellular signaling pathways in human breast cancer cell lines

**Figure 4:**
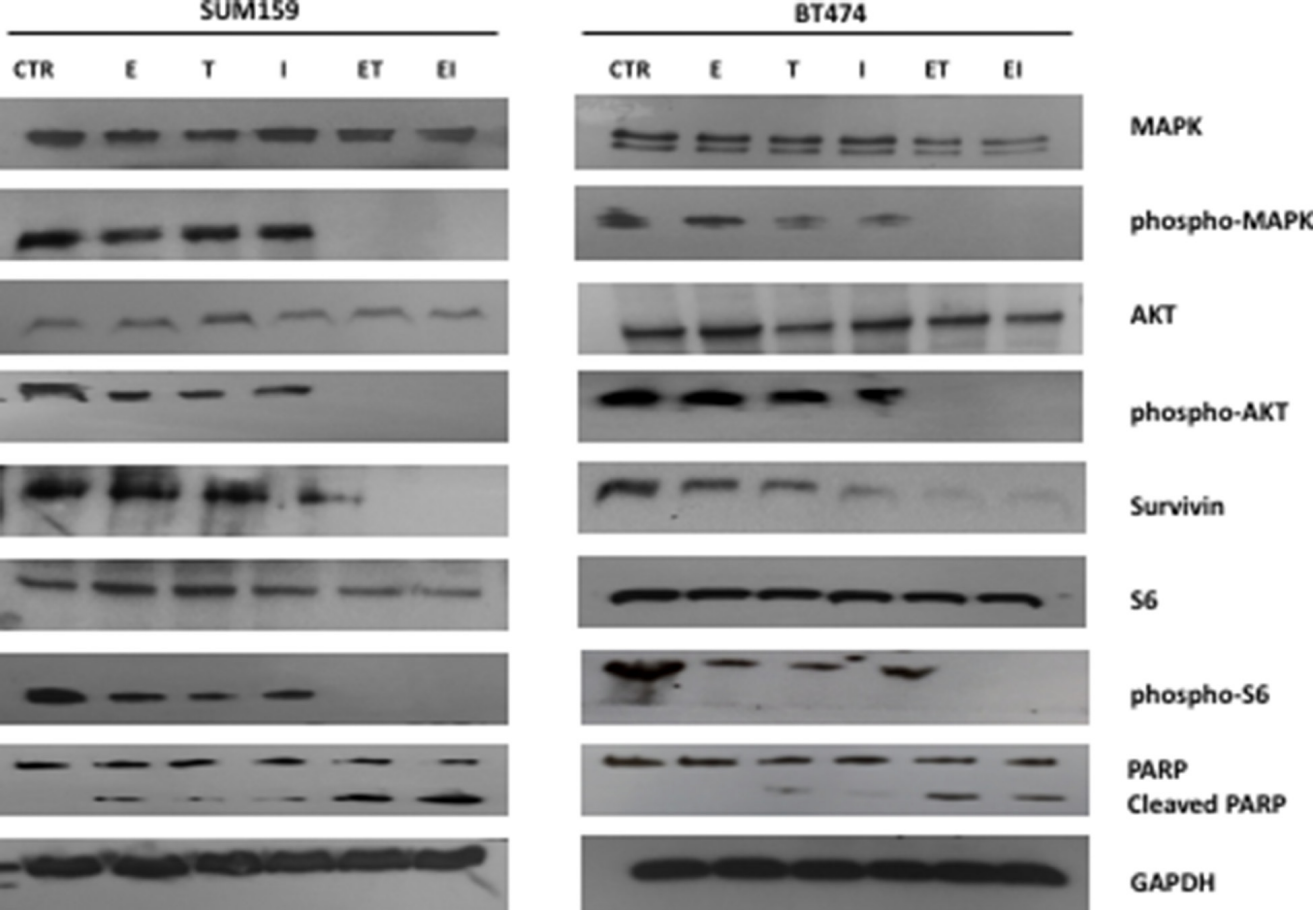
Effects of taselisib and ipatasertib treatment as single agent and combined with eribulin in BT474 and SUM159 cell lines on intracellular signaling pathways Western blotting analysis of intracellular proteins MAPK, AKT, S6, and their phosphorylated isoforms, survivin and PARP were performed on lysates from cells following indicated treatments. GAPDH was included as a loading control.

To study the effect on intracellular signaling pathways by the combination of taselisib and ipatasertib plus anti-microtubule chemotherapies, Western blot analyses were done on protein extracts from BT474 and SUM159 human breast cancer cells that were treated with taselisib and/or ipatasertib and eribulin (Figure 4). Treatments were conducted for 48 hours at the IC_50_ doses for cell growth inhibition. By investigating the effect of combined treatment on downstream proteins of PI3K pathway, we observed that eribulin, taselisib or ipatasertib treatment, as single agents, partially modified the activation of AKT and S6; only the combined treatments of taselisib or ipatasertib plus eribulin completely inhibited phosphorylation of AKT and S6 (Figure 4). Similarly, phosporylation of MAPK resulted significantly inhibited by the combined treatments (Figure 4). Of interest, survivin levels were partially reduced by single agent treatment and at a greater extent by the combined treatments both in SUM159 and BT474 cells (Figure 4). In addition, Western blot analysis for PARP protein (Figure 4) confirmed the results of apoptosis: the addition of taselisib or ipatasertib to eribulin treatment was able to induce the cleavage of the 113-kDa PARP to the 89-kDa fragments in sensitive breast cancer cell lines. Similar results were obtained with combined treatments with vinorelbine or paclitaxel plus taselisib or ipatasertib, also in KPL4 and MCF7 cells (data not shown).

### Effects of taselisib and ipatasertib plus anti-microtubule drugs on migration abilities of human breast cancer cell lines

**Figure 5:**
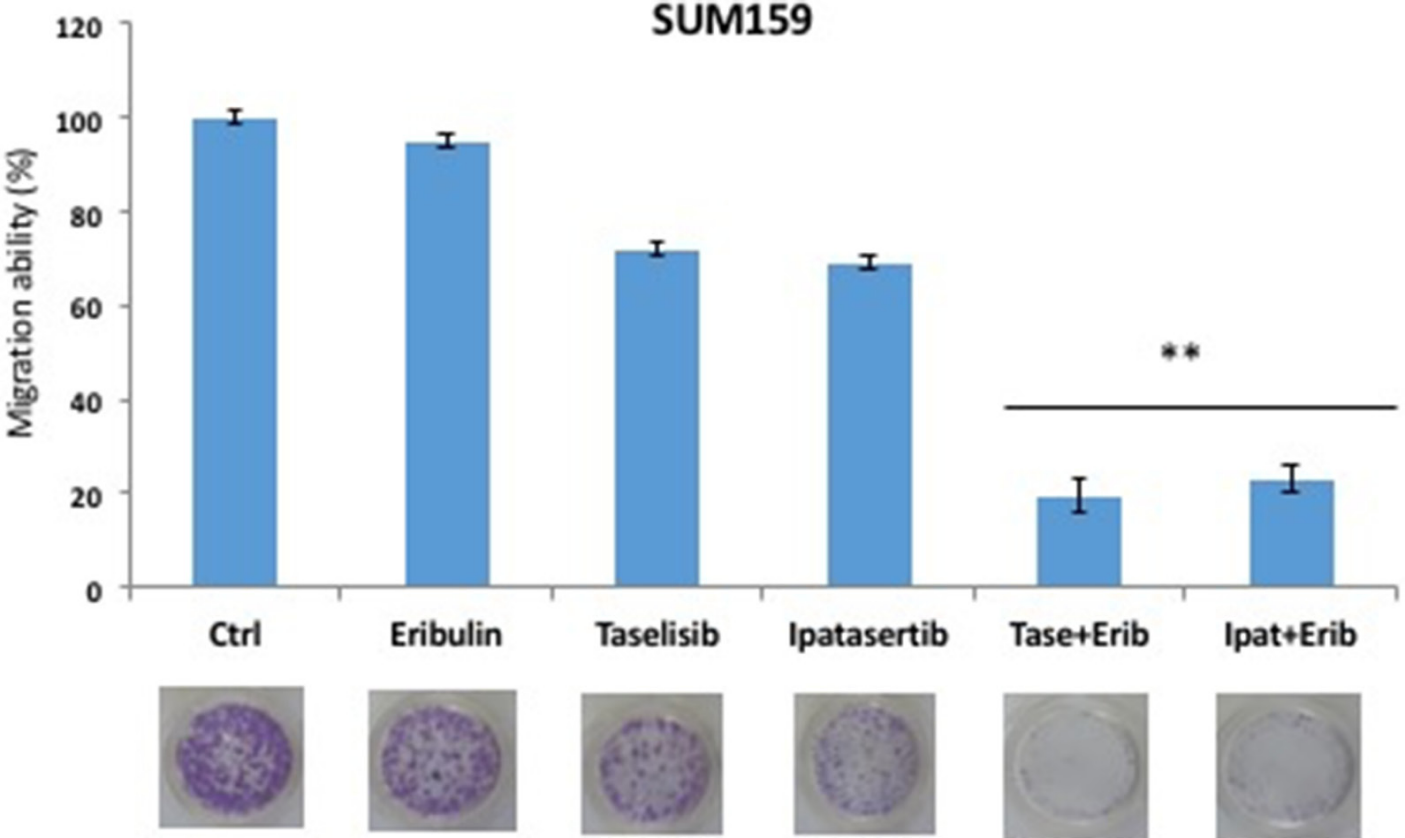
Effects on migration ability of taselisib and ipatasertib treatment as single agent and combined with eribulin in SUM159 cell line The results are the average ± SD of three independent experiments, each done in triplicate. *P* values < 0.01 were considered as statistically significant (**).

One important characteristic of malignant cells that depends also on their cytoskeleton organization is the ability to migrate, that can be measured *in vitro* as chemotactic properties. Among the panel of PI3Ka-mutated human breast cancer cell lines, SUM159 mesenchymal cells showed higher migration abilities. For this reason, SUM159 cells were treated with taselisib, ipatasertib or antimicrotubule drugs or with their combinations to study the effects on cell motility and migration. SUM159 cells were treated with taselisib, ipatasertib or eribulin, as single agents, or in combination. Treatment with IC_50_ doses for cell growth inhibition of taselisib, ipatasertib or eribulin had a little effect on the migration behaviour as compared to control cells treated with vehicle only (Figure 5). The addition of taselisib or ipatasertib to eribulin reduced to 19,5% and 23,2%, respectively, the migration ability of cancer cells as compared to vehicle or to single agent treatment (Figure 5).

### Effect of treatment with taselisib and ipatasertib in combination with anti-microtubule drugs on cytoskeleton network organization of human breast cancer cell lines

Considering the effect of anti-microtubule chemotherapic agents on cytoskeleton re-arrangement and the reduction of survivin protein levels observed with the combination treatments in Western blot analysis (Figure 4), we performed an immunofluorescence analysis in order to evaluate the effect of the combination treatment on cancer cell cytoskeleton dynamics.

**Figure 6:**
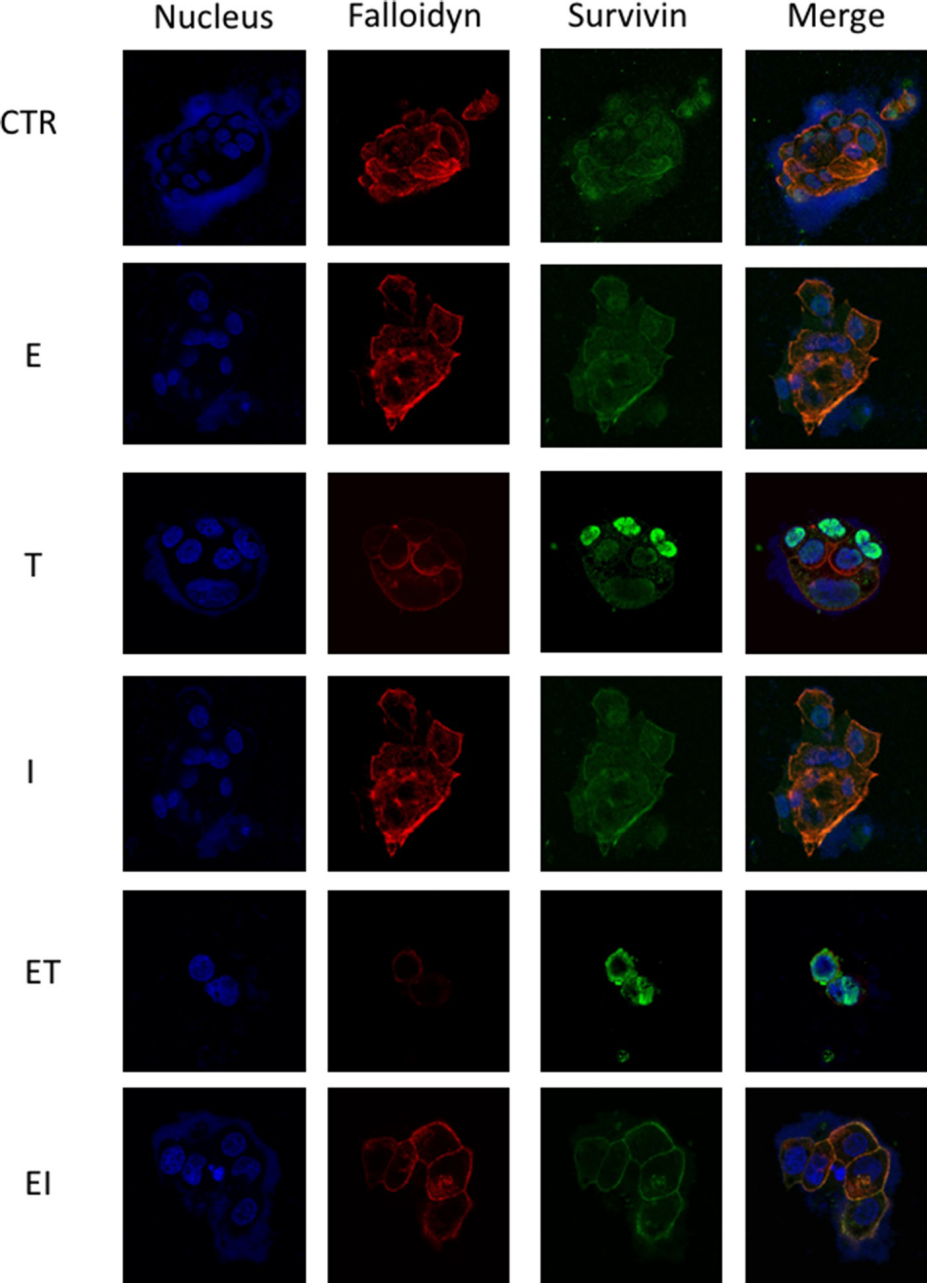
Effects of combined treatments with taselisib and ipatasertib plus eribulin on cytoskeleton organization Immunofluorescence staining for falloidyn and survivin, in BT474 cells, was done after the indicated treatments for 24 hours.

**Figure 7:**
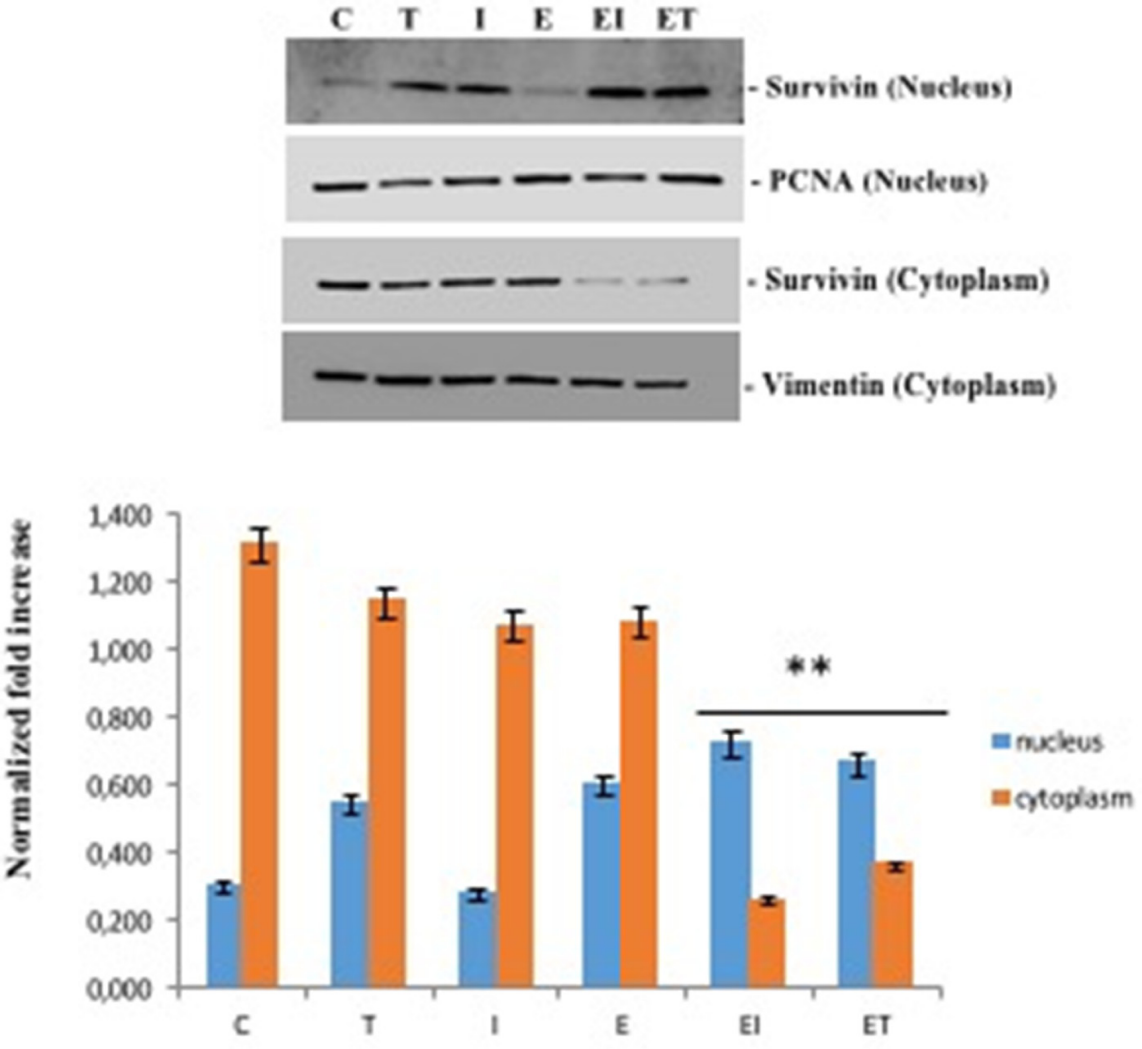
Effects of single agents taselisib and ipatasertib and combined treatments with eribulin on cytosolic and nuclear expression of survivin PCNA and vimentin were used as control for nucleus and cytoplasm, respectively. Separation of the two sub-cellular fractions of proteins was done as described in Matherials and Methods, after the indicated treatments for 24 hours in BT474 cells. *P* values < 0.01 were considered as statistically significant (**).

We performed an immunofluorescence staining for falloidyn, a marker of cytoskeleton organization, that binds specifically to F-actin and marks the actin stress fibers in the cell, and survivin, that modulates microtubule nucleation and polymerization, in BT474 human breast cancer cells after treatoment with eribulin, taselisib, ipatasertib or their combination for 24 hours (Figure 6). Single agent treatment with eribulin dysorganized microtubules, but only the addition of taselisib or of ipatasertib totally inhibited microtubules polimerization (Figure 6), suggesting a significant impairment of cytoskeleton organization by the combination treatment. To better investigate the mechanism of action of combined treatments, we analyzed nuclear and cytosolic fractions of cellular proteins (Figure 7). As compared to their respective control, survivin levels in cytosol decreased strongly with combination treatments eribulin plus taselisib or ipatasertib to 25,7% and 35,9% respectively; at the same time, nuclear levels of survivin increased from 30% to 72,2% and 66,2% in in presence of combined treatments, thus confirming the effect of these treatments in delocalizating survivin from cytoskeleton.

## DISCUSSION

PI3K/AKT signaling pathway plays a significant role in tumorigenesis, cancer survival and proliferation in human breast cancer [11]. PI3K/AKT activation often derives from mutation of the PI3Ka subunit, that is the catalytic subunit of the protein and responds to a variety of growth factors and integrin-mediated signals [11, 12], leading to AKT-induced intracellular cascade activation, that controls proliferation, survival, anti-apoptotis signals and microtubule dynamics. AKT becomes active by phosphorylation of two residues (T308 and S473), that is catalyzed by PDK1 and a protein complex called TORC2, respectively, which are regulated downstream of PI3K. The activation of AKT by phosphorylation (phospho-AKT) regulates critical cellular activities in many physiological contexts such as growth, proliferation, differentiation, metabolism, survival, as well as in pathological contexts, such as tumorigenesis and metastasis, by mediating growth factor stimulation of cell migration [11, 14, 26]. Active phospho-AKT plays an essential role in promoting cell migration in response to growth factors. It has also been shown that activation of Rac, as well as inactivation of the tumor suppressor gene PTEN, promotes migration of fibroblasts through activation of AKT. These data in part explain the invasiveness of malignant tumors with high AKT activity [14, 27].

PI3Ka and AKT mutations are detected in 30–50% of breast cancers [11, 12] and are associated with worse clinical outcomes in HR+ tumors [13], resistance to chemotherapy [13, 16], expecially to the anti-microtubule agent paclitaxel [28] and to anti-HER2 drugs [14], indicating that PI3K/AKT signaling activation can be clinically relevant. The Cancer Genome Atlas (TCGA) breast cancer analysis found PIK3CA mutation rates of 45% in luminal A, 29% in luminal B, 39% in HER2+, and 9% in the basal-like subtypes, respectively [29].

Thus, targeting PI3K pathway with specific inhibitors may represent a novel clinical strategy to switch off its oncogenic signal. Moreover, combinational strategies of anti-PI3K agents with other anti-tumoral agents could represent a new strategy to overcome intracellular escape mechanisms to conventional therapies. Various PI3K pathway inhibitors have now been evaluated in clinical trials [22, 30, 31]. Taselisib is a novel selective inhibitor of mutant PI3Kα that showed a potent acitivity in preclinical studies in different tumor types [19–21]. Taselisib is able in reducing tumor growth of mutant PI3Kα xenograft models of uterine serous carcinoma [19] and radiosensitizes PI3Kα mutated head and neck squamous carcinomas cells [21]. Furthermore, Hoeflich et al. demonstrated that treatment with taselisib is effective in reverting letrozole resistance in human breast cancer cells, by inhibition of PI3Kα hyperactivation [20]. The availability of these data speed up the clinical evaluation of combinational strategies including PI3K inhibitors and hormonal therapies. Taselisib is currently under clinical investigation in combination with hormonal therapy (NCT02340221, NCT02273973, NCT01296555) or taxanes (NCT01862081) in MBC.

Ipatasertib is a potent inhibitor of all three AKT isoforms, exhibiting a specific activity on mutant AKT1 [22–25]. AKT isoform 1 is involved in cellular survival pathways, by inhibiting apoptotic processes and in protein synthesis. Akt isoform 2 regulates glucose transport in insulin sensitive tissue. Akt isoform 3 have a yet unclear role. Ipatasertib showed dose-dependent inhibition of AKT signaling antitumor *in vitro* and *in vivo* in human tumor models with activated Akt signaling, representing a spectrum of cancer types, which includes prostate, breast, ovarian, colorectal, non-small cell lung, glioblastoma, and melanoma [22]. One clinical trial is currently evaluating the efficacy of ipatasertib in combination with paclitaxel in neoadjuvant (NCT02301988) setting of TNBC patients, while one phase II trial in first line (NCT02162719) therapy of TNBC patients, the LOTUS trial, has been recently concluded. Prelimary results of LOTUS trials, presented at last ASCO congress, demonstrated that the addition of ipatasertib to paclitaxel improved median progression free survival, expecially in patients harboured alterations of PIK3CA, AKT or PTEN, thus providing a clinical data for our experimental results.

In the present work, we focused on PI3Ka-mutated breast cancer models and we explored the efficacy of blocking AKT signaling trough direct inhibition of PI3Ka with taselisib or downstream inhibition of AKT protein with ipatasertib and their combination with the most widely used chemoterapeutic agents for MBC, the class of anti-microtubules (vinorelbine, eribulin, paclitaxel).

There are few data on a potential role of AKT inhibition as mechanism of action of the anti-microtubule agent paclitaxel [28], thus suggesting a rationale for this combinational strategy. In different breast cancer subtype cell models, all sharing high activity of AKT signal depending from PI3Ka activating mutation, including cells harbouring various expression profile of HER2 and HR receptors, we demonstrated a synergistic effect of combination of taselisib or of ipatasertib and anti-microtubule agents in terms of anti-proliferatve effect. The following question was whether this increased antiproliferative effect would be the result of an increased apoptosis. We demostrated that the blockade of PI3K/Akt pathway, which is hyper-activated in PI3Ka-mutated human breast cancer cells, concomitantly to the blockade of microtubules, enhances the apoptotic response as compared to single agent treatment. As there are evidences that PI3K and AKT have the potential to coordinately regulate microtubule organization and stability in a variety of tissues and cellular contexts [32], we further explored cancer cells migration ability, which is strictly dependent on cytoskeletal rearrangements and underlies the ability to metastatize. We have demonstrated that only combination treatments had the ability to reduce migration of cancer cells and, therefore, potentially to block metastatization. Moreover, Western blot analysis confirmed the inhition of PI3K pathway by single agent taselisib or ipatasertib but only their combination with eribulin, significantly affected phospho-MAPK and survivin levels. Immunofluorescence staining for falloydin and survivin allowed to further understand the mechanism of synergy of the combined treatment. First, combined treatment with anti-PI3K/AKT agents and eribulin dysrupted completely the organization of microtubules, thus confirming the potent mechanism of this combination in terms of anti-proliferative and pro-apoptotic effect. Second, treatment with single agent taselisib and at a greater extent with taselisib plus eribulin induced a de-localization of survivin from cytoplasm to peri-nuclear distribution, suggesting a potential interplay between PI3K signaling and survivin in the regulation of cellular cytoskeleton stability [33]. Combination treatments of taselisib and ipatasertib with eribulin induced delocalization of survivin from cytoplasm to nucleus, thus we speculate that inhibition of PI3K pathway may favour the activity of anti-microtubule agents, inducing the loss of survivin linking to cytoskeleton, through its traslocation to nucleus or maybe also favouring its degradation, as demostrated by reduced protein levels at western blot analysis (Figure 4) [33]. These effects on cytoskeleton organization may also explain the results obtained by the combined treatments on migration abilities of human breast cancer cells and represent an interesting issue for future investigations.

In conclusion, these results provide a biological rationale for further clinical evaluation of the anti-tumor efficacy of these combinations in human breast cancer patients.

## MATERIALS AND METHODS

### Cell lines

The human breast cancer cell lines MDA-MB231, MDA-MB438, BT474 and MCF7 cell lines were provided by American Type Culture Collection (ATCC, Manassas, VA, USA).

MDA-MB438 and MDA-MB231 were maintained in in Dulbecco's modified Eagle's medium (DMEM) supplemented with 10% fetal bovine serum (FBS; Life Technologies, Gaithersburg, MD, USA) and 1% antibiotics/antimycotics (Life Technologies, Gaithersburg, MD, USA).

BT474 were cultured in Roswell Park Memorial Institute (RPMI, Sigma-Aldrich, Saint Louis, Missouri, USA) medium added with 10% FBS and 1% antibiotics/antimycotics.

MCF7 were grown in the base medium ATCC-formulated Eagle's Minimum Essential Medium, (ATCC, Manassas, VA, USA - Catalog No. 30-2003), complemented by 01 mg/ml human recombinant insulin (Sigma-Aldrich, Saint Louis, Missouri, USA), 10% FBS and 1% antibiotics/antimycotics.

KPL4 cell line was kindly provided by Dr Roberto Bianco at Federico II University (Napoli, Italy) and cultivated in DMEM medium with 5% FBS and 1% antibiotics/antimycotics.

The SUM159 cell lines were purchased from Asterand Inc. (Detroit, MI, USA) and were grown in Ham's F-12 medium supplemented with 5% heat-inactivated fetal bovine serum (FBS) and 1% antibiotics/antimycotics (Invitrogen, Carlsbad, CA, USA).

All cell lines were maintained in a humidified incubator with 5% CO2 atmosphere at 37^°^C.

### Drugs

Paclitaxel was purchased from Selleck Chemicals (Selleckchem, Houston, TX, USA), Vinorelbine and eribulin from LookChem, Zhejiang J&C Biological Technology (China).

Taselisib and ipatasertib (GDC-0068) were provided by Genentech (Research proposal nr. OR-214726 for taselisib and nr. OR-214797 for ipatasertib).

They were dissolved in sterile dimethylsulfoxide (DMSO) and a 10 mM and 10 mM stock solutions were prepared and stored in aliquots at −20°C. Working concentrations were diluted in culture medium just before each experiment.

### Cell proliferation assays

Cancer cells were seeded in 96-well plates and were treated with different doses of indicated drudg for 72 hours as single agent or in combination. Constant ratio for combination was chosen considering the ratio between IC50 of each single drug. Cell proliferation was measured with the MTT assay, as previously described [34]. IC50 were determined by interpolation from the dose-response curves. Results represent the median of three separate experiments, each performed in quadruplicate. Synergism was calculated with CompuSyn software (ComboSyn Inc., Paramus, NK., USA).

### Colony forming assays

Cells were seeded on 6-well tissue culture dishes at 300 cells/well and treated with indicated drugs at IC50 doses. All conditions were performed in triplicate and untreated cells were used as control. Cells were maintained for 7 days at which point they were fixed with 4% paraphormaldeid, stained with crystal violet and colonies counted using the GelCount (Oxford Optronix, United Kingdom).

### Assessment of apoptosis

Apoptosis was evalued by flow citometry using an Annexin V/7AAD double staining (thermo fisher). Annexin V reveals the phosphatidylserine exposure of the altered plasma membrane; 7AAD is a vital dye, impermeant to live and apoptotic cells, and was used to distinguish death cells.

Briefly, cells were harvested, washed twice with PBS and then resuspended in 1X annexin-binding buffer at a concentration of 1 × 10^6^ cells/mL according to the manufacturer's instruction. Cells were stained adding 5 μL of Annexin V-FITC and 1 μL of 7AAD (100 μg/mL) into 100 μL of cell suspension and incubated in the dark for 15 min at room temperature. After the incubation, 400 μL of 1X annexin-binding buffer were added to the labeled cells and analyzed by flow cytometry within 30 min. All early apoptotic cells (Annexin V-positive, 7AAD–negative), necrotic/late apoptotic cells (double positive), as well as living cells (double negative) were detected by ACCURI C6 flow cytometer and subsequently analyzed by ACCURI C6 software (Becton Dickinson). Argon laser excitation wavelength was 488 nm; Annexin V-FITC was detected by FL-1 channel, 7AAD by FL-3 channel.

### Protein expression analysis

Following indicated treatments, cancer cells were lysed with Tween-20 lysis buffer (50 mmol/L HEPES, pH 7.4, 150 mmol/L NaCl, 0.1% Tween-20, 10% glycerol, 2.5 mmol/L EGTA, 1 mmol/L EDTA, 1 mmol/L DTT, 1 mmol/L phenylmethylsulfonylfluoride, and 10 μg/mL of leupeptin and aprotinin). Protein lysates containing equal amount of proteins, measured by a modified Bradford assay (Bio-Rad, Hercules, CA, USA), were subjected to Western blot analysis. Primary antibodies against p-MAPK44/42 (Thr202/Tyr204), MAPK44/42, p-AKT (Ser473), AKT, S6, p-S6 (Ser235-236), survivin, GAPDH and PARP were obtained from Cell Signaling Technology (Danvers, MA, USA). Secondary antibodies, goat anti-rabbit IgG and rabbit anti-mouse IgG, from Bio-Rad (Bio-Rad, Hercules, CA, USA) were used. Immunoreactive proteins were visualized by enhanced chemiluminescence (ECL plus; Thermo Fisher Scientific). Each experiment was done in triplicate.

### Migration assay

Cell migration was assessed using a commercially available chemotaxis assay. Briefly, cells were incubated in RPMI serum-free medium for 24 hours were left untreated or treated with the indicated treatments, following which they were detached from flasks, suspended in quenching medium (serum-free medium containing 5% bovine serum albumin) and EDTA, and seeded into Boyden migration chamber inserts placed in a 24-well plate (Cell Biolabs, CA, USA), containing a microporous membrane with an 8-μm pore size. Inserts were placed over wells containing serum-free media plus chemo-attractant (10% FBS). After a 48-h treatment period, cells/media were discarded from the topside of the migration chamber insert and the chamber was placed in the wells of a new 24-well plate containing cell detachment solution. Following incubation for 30 min at 37°C, the insert was discarded, and a solution of lysis buffer and CyQuant GR dye was added to each well (Invitrogen, OR, USA). CyQuant is a green fluorescent dye that exhibits strong enhancement of fluorescence when bound to cellular nucleic acids released by the lysis buffer, enabling assessment of the relative number of migrated cells. Fluorescence was determined with a fluorimeter at 480/520 nm. Assays were performed in triplicate.

### Immunofluorescence

For immunofluorescence assay on cancer cells, cells (3 × 10^4^/well) were plated in 24-well plates, previously covered with a glass cover slide (BD Biosciences, San Jose, CA, USA). After 24 hours of the indicated treatment, cells were fixed with freshly prepared 4% paraformaldehyde in PBS, permeabilized with 0.2% Triton X-100 for 5 min, and then rinsed three times with 1% bovine serum albumin in PBS (Sigma-Aldrich, Saint Louis, Missouri, USA). Cells were then incubated with the primary antibody Survivin (1:500) for 1 hour at room temperature (Cell Signaling Technologies, Danvers, MA, USA), and then with a fluorescent Cy2-AffiniPure Donkey Anti-Rabbit IgG secondary antibody (LiStarFish, Milan, Italy) for 1 hour at room temperature. Phalloidin-FITC (25 mg/ml) was obtained from Sigma-Aldrich (St Louis, MO, USA) and was used to visualize the actin cytoskeleton organization. Nuclei are stained in blue with Hoechst 33342 (1:25000) (Life Technologies, Gaithersburg, MD, USA) for 10 min at room temperature. Slides were mounted with 50% glycerol in PBS and imaged with a Zeiss LSM 510 meta confocal microscope (Zeiss, Oberkochen, Germany) equipped with an oil immersion plan apochromat _63 objective 1.4 NA.

### Cytosolic and nuclear protein extraction

Protein extraction to obtain separate cytosolic and nuclear fractions was also carried out. In detail, cells were homogenized in lysis buffer (10 mM Hepes pH 7.9, 0.1 mM EGTA pH 8.1, 1.5 mM MgCl_2_, 0.5 mM DTT, 12% glycerol) in presence of protease inhibitors [4 μg/ml leupeptin, aprotinin, pepstatin A, chymostatin, 5 μg/ml N-tosyl-Lphenylalanine chloromethyl ketone (TPCK), 0.5 mM phenylmethylsulfonylfluoride (PMSF)]. Homogenate was centrifuged at 4°C, 800 × *g* 10 min to pellet intact nuclei; supernatant containing soluble protein fraction was centrifuged again at 800 × *g*, thus pellet was added to the previous described pellet of intact nuclei and cleared supernatant was collected as cytosolic protein fraction and used for Western blot analysis. After that, pooled pellets of whole nucleus were homogenized in lysis buffer, containing in addition to the above described components, 0.420 M NaCl. After 30 min at 4°C on vortex, sample was centrifuged at 15,000 × *g* for 15 min thus to obtain a supernatant of nuclear proteins. PCNA (Santa Cruz Biotechologies, Dallas, TX, USA) and Vimentin (Cell Signaling Technology, Danvers, MA, USA) were used respectively as nuclear and cytosolic controls.

### Statistical analysis

The Student *t* test was used to evaluate the statistical significance of the results. All *P* values represent 2-sided tests of statistical significance.

## SUPPLEMENTARY MATERIALS FIGURES AND TABLES


