# Supplementary material for: Phosphatidylinositol 3-kinase (PI3Kα)/AKT axis blockade with taselisib or ipatasertib enhances the efficacy of anti-microtubule drugs in human breast cancer cells
Source: Oncotarget. 2017 Aug 22;8(44):76479–91. doi: 10.18632/oncotarget.20385 (PMC5652721; doi:10.18632/oncotarget.20385)
Supplement: Supplementary file 1 [file oncotarget-08-76479-s001.pdf]

Phosphatidylinositol 3-kinase (PI3Kα)/AKT axis blockade with taselisib or ipatasertib enhances the efficacy of anti-microtubule drugs in human breast cancer cells

SUPPLEMENTARY MATERIALS

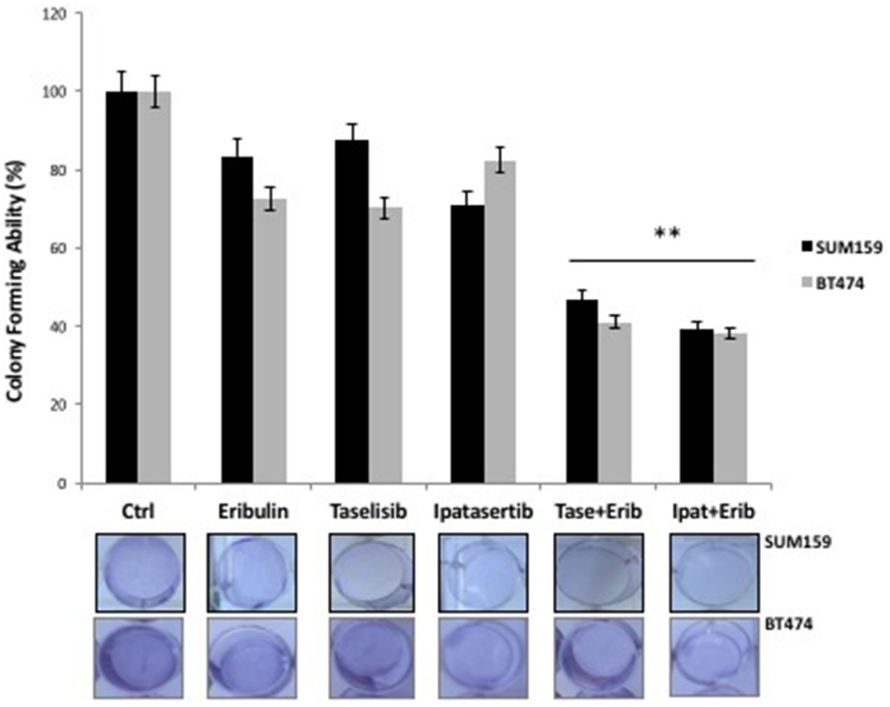

Supplementary Figure 1: Effects on colony forming assays of taselisib and ipatasertib treatment as single agents and combined with eribulin BT474 and SUM159 cell lines. Colony forming assays was performed as described in Materials and Methods, after the indicated treatments at IC50 doses. The results are the average ± SD of three independent experiments, each done in triplicate. *P* values < 0.01 were considered as statistically significant (**).

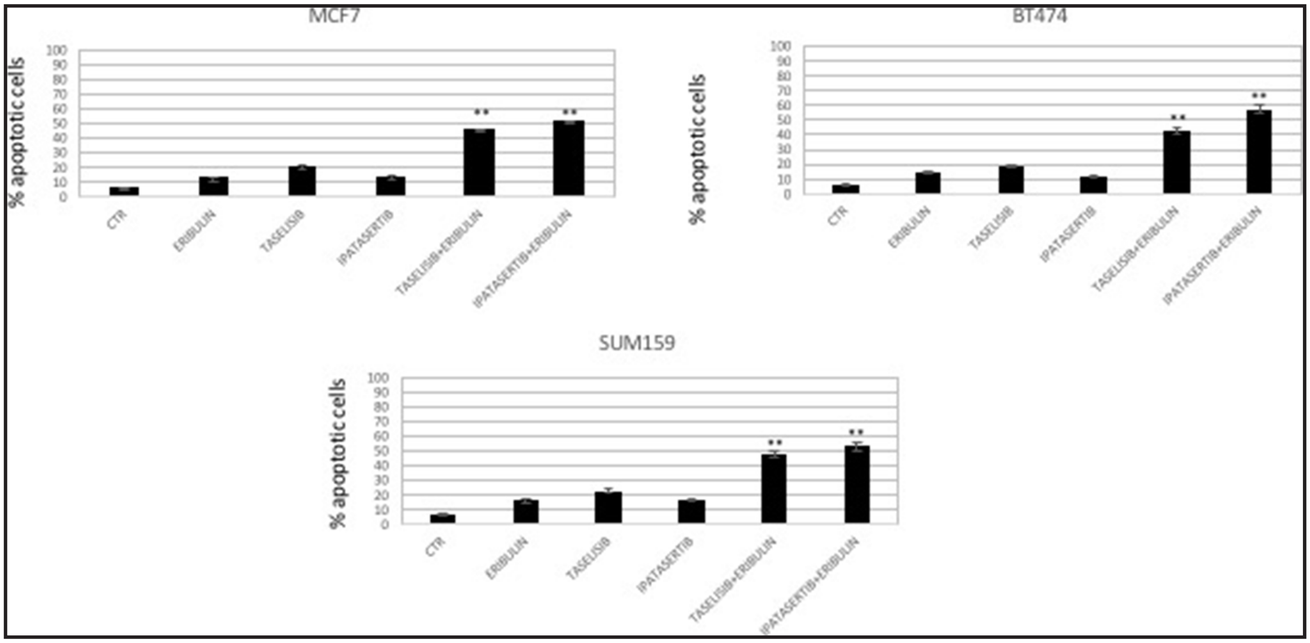

Supplementary Figure 2: Effects on apoptosis of taselisib and ipatasertib treatment as single agents and combined with eribulin in MCF7, BT474 and SUM159 cell lines. Apoptosis was evaluated as described in Materials and Methods with Annexin V staining in cancer cells, after the indicated treatments. Values of % apoptotic cells. Columns mean of 3 identical wells of a single representative experiment. *P* values < 0.01 were considered as statistically significant (**).
